# Primary Sources of Polycyclic Aromatic Hydrocarbons to Streambed Sediment in Great Lakes Tributaries Using Multiple Lines of Evidence

**DOI:** 10.1002/etc.4727

**Published:** 2020-06-11

**Authors:** Austin K. Baldwin, Steven R. Corsi, Samantha K. Oliver, Peter L. Lenaker, Michelle A. Nott, Marc A. Mills, Gary A. Norris, Pentti Paatero

**Affiliations:** ^1^ US Geological Survey Boise Idaho USA; ^2^ US Geological Survey Middleton Wisconsin USA; ^3^ US Environmental Protection Agency Cincinnati Ohio USA; ^4^ US Environmental Protection Agency Durham North Carolina USA; ^5^ Institute for Atmospheric and Earth System Research, University of Helsinki Helsinki Finland

**Keywords:** Polycyclic aromatic hydrocarbons, Sediment toxicity, Storm water runoff, Coal‐tar pavement sealant, Great Lakes, Positive matrix factorization

## Abstract

Polycyclic aromatic hydrocarbons (PAHs) are among the most widespread and potentially toxic contaminants in Great Lakes (USA/Canada) tributaries. The sources of PAHs are numerous and diverse, and identifying the primary source(s) can be difficult. The present study used multiple lines of evidence to determine the likely sources of PAHs to surficial streambed sediments at 71 locations across 26 Great Lakes Basin watersheds. Profile correlations, principal component analysis, positive matrix factorization source‐receptor modeling, and mass fractions analysis were used to identify potential PAH sources, and land‐use analysis was used to relate streambed sediment PAH concentrations to different land uses. Based on the common conclusion of these analyses, coal‐tar–sealed pavement was the most likely source of PAHs to the majority of the locations sampled. The potential PAH‐related toxicity of streambed sediments to aquatic organisms was assessed by comparison of concentrations with sediment quality guidelines. The sum concentration of 16 US Environmental Protection Agency priority pollutant PAHs was 7.4–196 000 µg/kg, and the median was 2600 µg/kg. The threshold effect concentration was exceeded at 62% of sampling locations, and the probable effect concentration or the equilibrium partitioning sediment benchmark was exceeded at 41% of sampling locations. These results have important implications for watershed managers tasked with protecting and remediating aquatic habitats in the Great Lakes Basin. *Environ Toxicol Chem* 2020;39:1392–1408. © 2020 The Authors. *Environmental Toxicology and Chemistry* published by Wiley Periodicals LLC on behalf of SETAC.

## Graphical abstract

Comparison of US Environmental Protection Agency 16 priority pollutant polycyclic aromatic hydrocarbon compound (ΣPAH_16_) concentrations in select sediments around the world. Bar = mean; whisker = maximum.

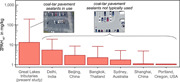

## INTRODUCTION

Representing 84% of the fresh surface water in North America (US Environmental Protection Agency 2015), the Great Lakes are an invaluable natural resource to the United States and Canada. However, a history of industrial, agricultural, and household pollution has left a legacy of contaminated sediments in many areas of the Great Lakes and their tributaries. Some of the contaminants, such as polychlorinated biphenyls (PCBs) and DDT, are primarily historical, because regulations in recent decades have resulted in major reductions in their source contributions. Other contaminants, such as polycyclic aromatic hydrocarbons (PAHs), have historical and modern sources, and therefore continue to enter and accumulate in the Great Lakes and their tributaries today (Baldwin et al. 2016, 2017). A recent study of organic compounds in water samples from Great Lakes tributaries found that among 15 classes of organic contaminants, including herbicides and insecticides, PAHs posed the greatest risk to aquatic organisms (Baldwin et al. 2016).

The PAHs are a class of >100 organic compounds composed of 2 or more fused aromatic rings. They are widespread contaminants with sources both historical and modern, and both natural and anthropogenic. Petrogenic PAHs, which form at low temperatures over geologic time scales, come from refined petroleum products (asphalt, diesel, gasoline, home heating oil, motor oil, and lubricants) and unprocessed coal and crude oil, among others (Pietara et al. 2010). Pyrogenic sources, in contrast, form at high temperatures during incomplete combustion of carbon‐based material (Pietara et al. 2010). Natural sources of pyrogenic PAHs include volcanic eruptions and wildfires. Anthropogenic sources of pyrogenic PAHs include residential wood burning, exhausts from diesel and gasoline engines, and emissions from coal‐fired power plants and coke‐ovens, creosote, and coal tar from pavement sealants and former manufactured gas plants (Mahler et al. 2005; Neff et al. 2005; Pietara et al. 2010).

A number of PAHs are carcinogenic, mutagenic, teratogenic, and/or toxic to aquatic organisms (Eisler 1987). As a result, the International Joint Commission has identified carcinogenic PAHs as a “critical pollutant” in the Great Lakes (Agency for Toxic Substances and Disease Registry 2008), making PAHs a priority for reduction and elimination efforts. The PAHs are listed as contaminants of concern at 61% of Great Lakes Areas of Concern (US Environmental Protection Agency 2013). To that end, the US Environmental Protection Agency (USEPA) and other groups have spent more than $550 million cleaning up contaminated sediment in the Great Lakes region since 2002, with a primary focus on PAHs, PCBs, and metals (US Environmental Protection Agency 2002). With such resources being devoted to sediment clean‐up, it is important to understand the distribution, magnitude, and sources of PAHs in the Great Lakes Basin.

The objectives of the present study were to 1) assess the occurrence and potential adverse biological effects of PAHs in recently deposited sediments in Great Lakes tributaries across 6 US states, and 2) identify the most important sources of PAHs to Great Lakes tributaries using multiple lines of evidence. The lines of evidence were 1) land‐use analysis, 2) parent/alkylated ratios, 3) high‐molecular‐weight/low‐molecular‐weight (HMW/LMW) ratios, 4) PAH profiles, 5) principal component analysis (PCA), 6) positive matrix factorization (PMF) receptor modeling, and 7) mass fraction analysis.

## MATERIALS AND METHODS

### Site selection

**Figure 1:**
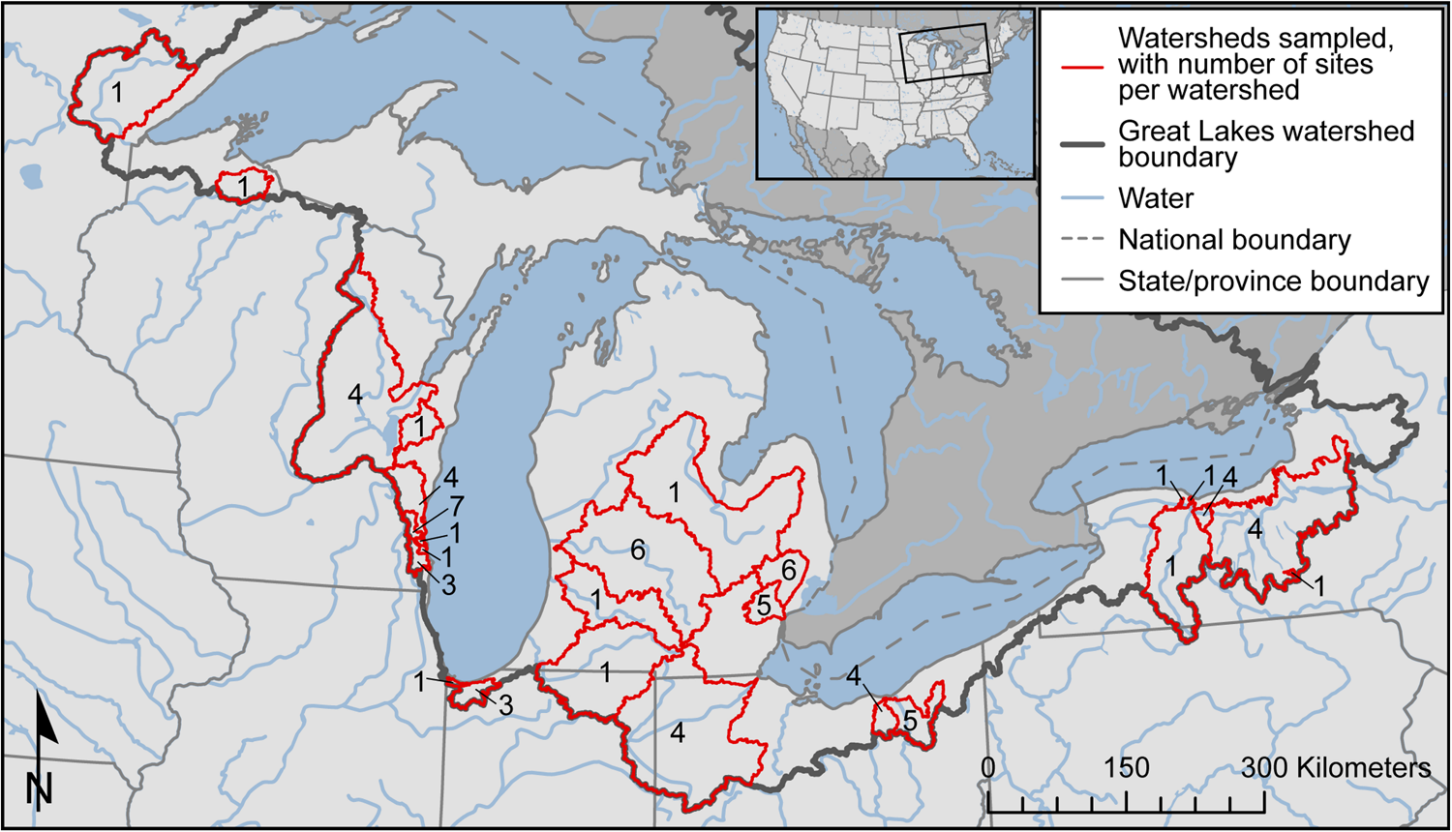
Map of the Great Lakes Basin and the watersheds sampled. Numbers indicate the number of sampling sites within each watershed.

Streambed sediment samples were collected from June to July 2017 from Great Lakes tributaries in Minnesota, Wisconsin, Michigan, Indiana, Ohio, and New York (USA). Between 1 and 7 locations within 26 tributary watersheds were sampled for a total of 71 sampling locations (Table 1, Figure 1, and Supplemental Data, Table S1). Locations were selected to represent watersheds with a range of land uses, and were from 0.7 to 100% urban. Watershed drainage areas ranged from 3.5 to 16 300 km^2^, and population densities ranged from 2.8 to 2260 persons/km^2^.

**Table 1:** Sampling locations and basin statistics^a^

Lake	Watershed	Site name	Site abbreviation	% Impervious	Drainage area (km^2^)	Population density (people/km^2^)
Erie	Clinton	Clinton River at Sterling Heights, MI	MI‐CLT	16	803	443
		Red Run at Ryan Rd nr Warren, MI	MI‐RRR	52	89	1734
		Bear Cr Immediately DS at Miller Drain at Warren, MI	MI‐BAR	72	48	1518
		Red Run at 15 Mile Rd at Sterling Heights, MI	MI‐RRS	53	275	1609
		North Branch Clinton River nr Mt. Clemens, MI	MI‐NBC	3.7	512	84
		Clinton River at Moravian Dr at Mount Clemens, MI	MI‐CRM	21	1937	611
	Cuyahoga	Cuyahoga River at Old Portage, OH	OH‐CRP	9.3	1047	297
		Cuyahoga River at Independence, OH	OH‐CRI	11	1836	326
		West Cr at Independence, OH	OH‐WCI	28	35	1130
		Cuyahoga River at Munroe Falls, OH	OH‐CRM	5.1	841	159
		Tinkers Cr at Dunham Rd nr Independence, OH	OH‐TCD	20	246	462
	Maumee	Maumee River at Waterville, OH	OH‐MRW	2.4	16 295	54
		Swan Cr at Toledo, OH	OH‐SCT	6.9	519	174
		Swan Cr at Oak Openings Metropark, OH	OH‐SCO	2.3	232	57
		Swan Cr at Township Road EF nr Swanton, OH	OH‐SCE	2.0	65	49
	Rocky	West Branch Rocky River nr Medina, OH	OH‐WBR	10	158	323
		Rocky River nr Berea, OH	OH‐RRB	9.5	692	358
		Rocky River above STP nr Lakewood, OH	OH‐RRS	11	755	408
		East Branch Rocky River at W Center St, Berea, OH	OH‐EBR	10	193	441
	Rouge	River Rouge at Birmingham, MI	MI‐RRB	24	95	658
		River Rouge at Detroit, MI	MI‐RRD	34	476	965
		Lower River Rouge at Beck Rd nr Sheldon, MI	MI‐LRB	7.9	24	242
		Lower River Rouge at Haggerty Rd at Wayne, MI	MI‐LRH	16	95	376
		Lower River Rouge at Wayne Rd at Wayne, MI	MI‐LRW	23	183	595
Huron	Saginaw	Saginaw River at Saginaw, MI	MI‐SAG	3.0	15 509	69
Michigan	Burns Ditch	Portage‐Burns Waterway at Portage, IN	IN‐PBW	14	857	345
		Coffee Cr DS of 1100 N nr Chesterton, IN	IN‐CCU	3.4	32	68
		Coffee Cr at Chesterton, IN	IN‐CCD	6.4	40	122
	Fox	Garners Cr at Park Street at Kaukauna, WI	WI‐GCK	30	21	834
		East River below Cedar St at Green Bay, WI	WI‐ERG	7.1	381	200
		West Branch Mud Cr below CTH BB at Appleton, WI	WI‐WMC	17	26	175
		Ashwaubenon Cr above Parkview Rd at De Pere, WI	WI‐ACA	10	75	106
	Grand	Peacock Ditch at Grand River Ave nr Ionia, MI	MI‐PEA	1.5	15	9.0
		Indian Mill Cr at Turner Ave at Grand Rapids, MI	MI‐IND	16	44	297
		Plaster Cr at 28th St at Grand Rapids, MI	MI‐PLS	27	119	468
		Tributary to Buck Cr at Division Ave at Wyoming, MI	MI‐TBC	48	16	1396
		Buck Cr at State Hwy M‐21 at Grandville, MI	MI‐BCK	30	131	761
		Grand River at Eastmanville, MI	MI‐GRE	4.3	13 560	109
	Indiana Harbor Canal	Indiana Harbor Canal at East Chicago, IN	IN‐IHC	47	100	914
	Kalamazoo	Kalamazoo River at New Richmond, MI	MI‐KAL	3.5	5122	91
	Manitowoc	Manitowoc River at Manitowoc, WI	WI‐MAM	1.6	1343	25
	Milwaukee	Milwaukee River at Milwaukee, WI	WI‐MIE	6.0	1785	195
		Milwaukee River at Mouth at Milwaukee, WI	WI‐MIM	12	2240	434
		Milwaukee River at Walnut St at Milwaukee, WI	WI‐MIP	6.5	1804	233
		Northridge Lake nr Milwaukee, WI	WI‐NRL	49	3.5	1441
	Menomonee	Menomonee River at CTH F nr Germantown, WI	WI‐MEF	2.3	29	67
		Menomonee River at Butler, WI	WI‐MEB	18	154	387
		Little Menomonee River at Lovers Ln at Milwaukee, WI	WI‐LML	19	55	634
		Menomonee River above Church St at Wauwatosa, WI	WI‐MEC	23	288	579
		Menomonee River nr N 25th St at Milwaukee, WI	WI‐MET	28	355	966
		Menomonee River at Ridge Blvd at Wauwatosa, WI	WI‐MER	21	233	525
		Underwood Cr at Juneau Blvd at Elm Grove, WI	WI‐UCJ	21	23	520
	Kinnickinnic	Kinnickinnic River at Lincoln Ave at Milwaukee, WI	WI‐KKL	51	62	2265
	Oak	Oak Cr at Mill Pond at South Milwaukee, WI	WI‐OCM	31	69	739
	Root	Root River at Layton Ave at Greenfield, WI	WI‐RRL	32	31	1150
		Root River nr Franklin, WI	WI‐RRR	25	127	830
		Root River nr Clayton Park at Racine, WI	WI‐RRC	12	506	334
	St. Joseph	St. Joseph River at Niles, MI	MI‐SJO	3.8	9628	80
Ontario	Cascadilla	Cascadilla Cr at Ithaca, NY	NY‐CCI	2.3	37	150
	Genesee	Genesee River at Ford St Bridge, Rochester, NY	NY‐GRF	1.2	6403	45
	Irondequoit	Irondequoit Cr at Railroad Mills nr Fishers, NY	NY‐ICR	2.4	100	78
		Allen Cr Near Rochester, NY	NY‐ACR	18	80	758
		Irondequoit Cr above Blossom Rd nr Rochester, NY	NY‐ICB	8.9	364	442
		Thomas Cr at East Rochester, NY	NY‐TCR	5.4	74	367
	Northrup	Northrup Cr at North Greece, NY	NY‐NCG	5.6	26	294
	Oswego	Harbor Brook at Hiawatha Blvd, Syracuse, NY	NY‐HBK	16	31	782
		Geddes Brook at Fairmount, NY	NY‐GBF	15	22	594
		Ley Cr at Lemoyne and Factory at Mattydale, NY	NY‐LEY	34	62	812
	Slater	Slater Cr at Hojack Industrial Pk at Mount Read, NY	NY‐SCH	25	12	1610
Superior	Bad	Bad River nr Odanah, WI	WI‐BRO	0.2	1545	2.8
	Saint Louis	Saint Louis River at Scanlon, MN	MN‐SLR	0.5	8890	9.2

^a^ Sites are ordered upstream to downstream within each watershed.

MI = Michigan; OH = Ohio; IN = Indiana; WI = Wisconsin; NY = New York; MN = Minnesota; nr = near; DS = downstream; Cr = Creek; STP = Sewage treatment plant; W = West; N = North; St = Street; Rd = Road; Dr = Drive; Ave = Avenue; Ln = Lane; Blvd = Boulevard; Hwy = Highway; Pk = Park; CTH = County Trunk Highway.

### Streambed sediment sample collection and analysis

Sample collection and analysis methods are detailed in the Supplemental Data. To summarize, streambed sediment sample collection was performed either by boat or while wading in the stream. Fine‐grained sediments (silts) were targeted at all locations. Sediment was collected to a depth of 15 cm using a push core sampler (WaterMark® Universal Core Head Sediment Sampler, Forestry Suppliers) with a 70‐mm (2 3/4 inch) outer diameter, a 66.7‐mm (2 5/8 inch) inner diameter, and 1.6‐mm (1/16 inch) wall polycarbonate tubing (Forestry Suppliers; or United States Plastic). The depth of 15 cm was selected to focus on recently deposited sediments, and thus modern versus historical PAH sources. The sediment was emptied into a stainless‐steel pan and split vertically; then one‐half was transferred to a baked amber‐glass bottle for use in the present study. The other half was used for a separate study that is not described in the present study. Samples were stored in the dark on ice. All sediment processing equipment was field‐cleaned between sampling locations by scrubbing with detergent (Alconox®) water followed by 3 rinses each of tap and deionized water. A new core tube was used at each location. Within 48 h of collection, samples were shipped to the Battelle Memorial Institute (Norwell, MA, USA) for analysis of 18 parent (the 16 USEPA priority pollutant PAHs [ΣPAH_16_] plus perylene and benzo[*e*]pyrene) and 18 alkylated PAHs (Supplemental Data, Table S2) via gas chromatography mass–spectrometry operated in selected ion monitoring mode. A split of the sample was sent to ALS Environmental (Kelso, WA, USA) for total organic carbon (TOC) analysis (modified from ASTM International [2005] method D 4129‐05).

Laboratory detection limits for PAHs ranged from 0.17 to 70.5 µg/kg (median 0.48 µg/kg), varying by compound and by analytical batch. The detection limit for TOC was 0.05%. Only 5.4% of PAH results were below the detection limit, and no TOC results were below the detection limit. Zeros were used as conservative substitutes for PAH concentrations below the detection limit in summations of total sample concentrations.

Duplicate samples were collected at 8 locations, resulting in 288 duplicate pairs (8 duplicate pairs for each of the 36 compounds). The PAH concentrations in 22 of the duplicate pairs were below the detection limit in both samples; in 4 duplicate pairs, concentrations were below the detection limit in 1 of the 2 samples. Relative percentage differences (RPDs) were calculated for all remaining duplicate pairs (i.e., those with concentrations above the detection limit in both samples). The median RPD was <20% for 24 of the 36 PAH compounds. The compounds with the highest RPDs (medians of 20.7–42.4%) were generally those with the lowest molecular weights and occurring at the lowest concentrations (parent and alkylated naphthalene, parent and alkylated fluorene, acenaphthylene, and acenaphthene). The TOC duplicates (*n* = 5) had RPDs < 4%.

Field blanks were collected at 5 locations by pouring organic‐free water (OmniSolv®) through a core tube into the stainless‐steel pan, and then into a baked amber‐glass jar. The majority of field blank PAH concentrations (71%) were below the detection limit; the 29% of blank concentrations above the detection limit ranged from 0.30 to 5.20 ng/L. There were no instances of blank concentrations above the detection limit when accompanying environmental sample concentrations were below the detection limit. All TOC blanks (*n* = 5) were below the detection limit. Results of laboratory blanks, spikes, surrogates, and duplicates are summarized in the Supplemental Data.

### Predicted toxicity using sediment quality guidelines

Potential PAH‐related toxicity of streambed sediments to aquatic organisms was assessed using the following sediment quality guidelines: the probable effect concentration (PEC; 22 800 µg/kg for ΣPAH_16_), the threshold effect concentration (TEC; 1610 µg/kg for ΣPAH_16_), and the sum equilibrium‐partitioning sediment benchmark toxicity unit (ΣESBTU; Ingersoll et al. 2001; US Environmental Protection Agency 2003; Kemble et al. 2013). The PEC quotients (PECQs) and the TEC quotients (TECQs) were computed for each sample by dividing the ΣPAH_16_ concentration in the sample by the PEC and TEC, with adverse effects to benthic organisms predicted at PECQs > 1.0, and unlikely at TECQs < 1.0 (Ingersoll et al. 2001).

The ΣESBTU approach accounts for the biological availability of individual PAH compounds in a mixture, and is applicable across sediment types (US Environmental Protection Agency 2003). To compute the ΣESBTU, TOC‐normalized concentrations of 35 PAHs (listed in the Supplemental Data, Table S2) were divided by compound‐specific final chronic values and summed. Streambed sediments with ΣESBTUs < 1.0 are expected to be nontoxic to benthic organisms from PAHs, whereas sediments with ΣESBTUs > 1.0 are expected to have adverse effects from PAHs.

Toxicity related to contaminants other than PAHs was not considered in the present study.

### Geographic information system methods

Watershed boundaries were determined in a geographic information system (GIS) for each site, using linework from the Watershed Boundary Dataset and catchments from the medium‐resolution NHDPlus V2 Dataset (US Department of Agriculture‐Natural Resources Conservation Service et al. 2009; US Environmental Protection Agency and US Geological Survey 2012). Resulting site watershed boundaries were used to summarize various watershed characteristics using tools available in the National Water‐Quality Assessment (NAWQA) Area‐Characterization Toolbox (Price 2017), which allows for standard GIS summaries in nested basins (Price 2017). Specifically, mean 2011 percentage imperviousness and mean 2012 parking lot abundance were calculated using the Feature Statistics to Table tool, and 2012 land‐use category percentages were determined using the Tabulate Features to Percent tool (Falcone 2015; Homer et al. 2015; Falcone and Nott 2019). Land‐use data were further summarized into major areas: transportation (code 21 in the NAWQA Anthropogenic Land Use Trends Dataset; Falcone 2015), commercial (code 22), industrial/military (code 23), residential (codes 25–26), and total urban (codes 21–27). Notably, the calculated parking lot abundance in each basin was derived from (and therefore overlaps with) the land‐use categories. For example, an area categorized as commercial likely includes (and does not exclude) adjacent parking areas.

Watershed population summaries were determined by creating a mosaic of 2010 state census block polygons, calculating the population density/census block polygon, intersecting census block and watershed boundaries, calculating the areas of the intersected polygons, multiplying these areas by the population density, and summing the result by watershed (US Census Bureau Geography Division 2010a, 2010b, 2010c, 2010d, 2010e, 2010f, 2010g).

### Identification of PAH sources

Multiple lines of evidence were used to identify the most likely source of PAHs to sediment samples. This approach mitigates uncertainties of individual methods and strengthens the overlapping conclusions (Larsen and Baker 2003; O'Reilly et al. 2014). The individual methods used were described previously (Baldwin et al. 2017), and are briefly described in the present study. Many of the methods used a subset of the 36 PAHs analyzed. The number of PAHs used and the reason for subsetting are described for each method.

### Land‐use analysis

Relations between different urban land uses and streambed sediment ΣPAH_16_ concentrations were assessed by using Spearman correlation with a significance level (*p* value) of 0.05. Spearman correlation results were unaffected by treatment of results below the detection limit: substitutions with 0, ½ × detection limit, and 1 × detection limit were each tested, and all yielded the same correlation values.

### Parent/alkylated and HMW/LWM compounds

Ratios of the mass concentrations of parent and alkylated PAHs were used to differentiate between petrogenic and pyrogenic sources. Petrogenic sources are generally dominated by alkylated compounds, whereas pyrogenic sources are generally dominated by parent compounds (Neff et al. 2005). Parent/alkylated ratios were computed using only the compounds for which both the parent and alkylated forms were measured. The 8 parent compounds included for this analysis were benz[*a*]anthracene, chrysene, pyrene, fluoranthene, fluorene, naphthalene, anthracene, and phenanthrene. The 18 alkylated compounds were the C1 to C3 or C1 to C4 alkylated forms of the 8 parents (Supplemental Data, Table S2).

Ratios of the mass concentrations of LMW (2–3 rings) and HMW (>3 rings) PAHs (Supplemental Data, Table S2) were also used to differentiate between petrogenic and pyrogenic sources. Petrogenic sources are generally dominated by LMW compounds, whereas pyrogenic sources are generally dominated by HMW compounds (Crane 2014).

### PAH profiles

A PAH profile was computed for each streambed sediment sample using the proportional concentrations of 12 PAHs (in order of increasing molecular weight): phenanthrene, anthracene, fluoranthene, pyrene, benz[*a*]anthracene, chrysene, benzo[*b*]fluoranthene, benzo[*k*]fluoranthene, benzo[*e*]pyrene, benzo[*a*]pyrene, indeno[1,2,3‐*cd*]pyrene, and benzo[*ghi*]perylene. The proportional concentration of each compound was calculated as the fraction of the ΣPAH_12_ concentration; each 12‐compound profile was summed to 1.0. Samples with concentrations below the detection limit were excluded from analysis of PAH profiles (and PCA, described in the *PCA* section following) because concentrations of all compounds are necessary to properly characterize the PAH profiles for these analysis methods.

The sample profiles were compared quantitatively with 12‐compound proportional concentration profiles of different sources from the literature (Table 2 and Supplemental Data, Table S3). Benzo[*e*]pyrene was not reported in the profiles of creosote‐treated railway ties (representing weathered creosote) and creosote product (unweathered). For those profiles, following Li et al. (2003), the benzo[*e*]pyrene concentration was assumed to be the same as that of benzo[*a*]pyrene. The similarity between source and sample profiles was evaluated using the chi‐square (χ^2^) statistic, calculated as the square of the difference in proportional concentrations of individual compounds, divided by the mean of the 2 values, summed for the 12 PAHs (Van Metre and Mahler 2010). A lower χ^2^ indicates greater similarity between source and sample profiles. A profile was not computed for one site, Kalamazoo River at New Richmond, MI (MI‐KAL), because of concentrations below the detection limit.

**Table 2:** Polycyclic aromatic hydrocarbon (PAH) sources used in PAH profiles, principal component analysis, and positive matrix factorization model

PAH source category	PAH source	Abbreviation
Coal combustion	Power plant emissions^a^	PPLT
	Coal average^a^	CCB1
	Residential heating^a^	RESI
	Coke oven emissions^a^	COKE
Vehicle related	Diesel vehicle particulate emissions^a^	DVEM
	Gasoline vehicle particulate emissions^a^	GVEM
	Traffic tunnel air^a^	TUN1
	Vehicle/traffic average^a^	VAVG
	Tire particles^a^	TIRE
	Used motor oil #1^a^	UMO1
	Used motor oil #2^a^	UMO2
Plant combustion	Pine wood soot particles #1^a^	PIN1
	Pine wood soot particles #2^b^	PIN2
	Oak wood soot particles^b^	OAKS
Coal tar	Coal‐tar pavement sealant product^c^	CTR0
	Coal‐tar–sealed pavement dust, 7 city average^d^	CTD7
Creosote	Creosote product^e^	CRE4
	Creosote‐treated railway ties, weathered^f^	CRE2
Miscellaneous	Fuel‐oil combustion particles^a^	FOC1
	Asphalt^a^	ASP2

^a^ Van Metre and Mahler 2014.

^b^ Crane 2014.

^c^ Van Metre et al. 2012.

^d^ Baldwin et al. 2017.

^e^ Neff 2002.

^f^ Covino et al. 2016.

### PCA

The PCA was performed using the same 12‐compound PAH profiles just discussed, with data standardized to have a mean of 0 and unit variance. Euclidean distances in *n*‐dimensional space were computed between sources and samples in the space defined by the principal components that accounted for ≥10% of the variability. Sources with the shortest Euclidean distance to the samples were considered to be most similar to the samples. The PCA computation was done using the prcomp function from the stats package in R (R Core Development Team 2015).

### PMF receptor model

The PMF is a multivariate receptor modeling tool that decomposes a matrix of speciated sample data into 2 matrices—sample contributions and factor profiles (Norris et al. 2014; Brown et al. 2015). The theory and detailed methods of PMF have been described previously (Paatero and Tapper 1994; Paatero 1997; Norris et al. 2014). The PMF was run using USEPA PMF Ver 5.0.14, a graphical user interface for the Multilinear Engine Program ME‐2. Unlike receptor models based on weighted linear regression (e.g., the USEPA Chemical Mass Balance Model), which require selection of the sources contributing to a sample prior to the regression analysis, the USEPA PMF determines profiles based on sample concentrations and the number of sources or *factors* specified by the user. The PMF does not identify the factor names; it is up to the user to match the factor profiles to known sources using measured source profiles employing approaches like the chi‐square analysis we describe.

A strength of the PMF model is that it considers the uncertainty of individual compound concentrations and the total sample concentration. The uncertainty of individual compound concentrations was computed using Equation 1 (Qi et al. 2016):

(1) $$\text{Uncertainty}=\sqrt{{\left(\mathrm{error}\times \mathrm{concentration}\right)}^{2}+{\mathrm{DL}}^{2}}$$

where *DL* is the detection limit. The *error* term (i.e., measurement error) was calculated as the median relative percentage difference between duplicate samples. The error term was therefore specific to each compound, varying from 0.12 to 0.20. The uncertainty on the total sample concentration (the combined standard uncertainty, *U*_*c*_(*x*)) was computed as the root sum of the squares of the individual compound uncertainties (Equation 2):

(2) $$U_{c}(x)=\sqrt{\left(u_{1}{\left(x\right)}^{2}+u_{2}{\left(x\right)}^{2}+u_{3}{\left(x\right)}^{2}+\;\text{⋯}\right)}$$

No extra modeling uncertainty was included in the PMF analysis.

The model was run in robust mode with 29 compounds (*species*), all of which were considered “strong” based on signal‐to‐noise ratios > 1.0 (concentrations were ≥2 × uncertainties). Two compounds, C4‐chrysenes and C3‐fluorenes, were omitted from the PMF analysis because of low detection frequencies. In addition, naphthalene and its alkylated forms (C1–4) were omitted because they were poorly predicted by PMF (observed vs predicted *r* ^2^ values of 0.004–0.23), likely because of their low molecular weight and high volatility. The omission of naphthalenes reduced (i.e., improved) the model's *Q* value (a goodness‐of‐fit parameter) by 1344. A total sample concentration variable was also included as a species but was downweighted to “weak” (Norris et al. 2014).

Three samples (Indiana Harbor Canal at East Chicago, Indiana [IN‐IHC], Geddes Brook at Fairmount, NY [NY‐GBF], and Cuyahoga River at Independence, OH [OH‐CRI]) were omitted from the PMF analysis because their unique PAH sources were not similar to the other samples. These sites had high scaled residuals for one or more PAHs and/or abnormally high concentrations. Samples with one or more concentrations below the detection limit were excluded from the PMF analysis for consistency with other methods used in the present study (i.e., profile correlations and PCA), which resulted in 14 samples being omitted. A total of 54 samples were included in the final PMF runs. The final PMF input files are provided in the Supplemental Data, Tables S4 and S5.

Multiple PMF runs were performed to determine the best solution, varying the number of factors (2–4) and the convergence criteria (default vs relaxed; see the Supplemental Data for discussion of relaxed convergence criteria) between runs. Each run consisted of 50 base runs with a random seed value. The best solution within each run was the one that minimized *Q*_robust_, a goodness‐of‐fit statistic calculated by dynamically downweighting points for which the uncertainty‐scaled residual was >4.0 (Paatero 1997). The solution was then tested for rotational ambiguity (the existence of multiple similar solutions, which may invalidate the chosen solution) using the USEPA PMF's displacement analysis, and for disproportionate effects of a small set of observations using the USEPA PMF's bootstrapping analysis. Bootstrapping was performed with 100 runs, with a block size of 2 and a minimum *r* value of 0.6. The final solution was the one that maximized the number of factors to account for the most important sources while still minimizing rotational ambiguity and disproportionate effects of small sets of observations. The final solution was further tested for rotational ambiguity by adding constraints using ratios derived from the 12‐compound source profiles from the literature (Supplemental Data, Table S3). Constraints are further discussed in the Supplemental Data. Finally, to assess potential bias in the goodness‐of‐fit toward low or high concentration samples, scaled residuals were plotted against ΣPAH_29_.

To match each of the PMF factors to a specific PAH source, the factors were compared with source profiles from the literature (Table 2 and Supplemental Data, Table S3) using the χ^2^ statistic. Although PMF was run using 29 compounds, the χ^2^ was computed using only the 12 compounds available in the literature source profiles.

**Table 3:** Sum concentration of US Environmental Protection Agency 16 priority pollutant polycyclic aromatic hydrocarbon compounds (ΣPAH_16_) for different sources

		ΣPAH_16_ concentrations (µg/kg)		
Type	PAH sources (no. of samples)	Mean	Maximum	Reference(s)
Particulates	Creosote‐treated wood (7)	63 365 000	97 181 000	Marcotte et al. 2014; Covino et al. 2016
	CT sealant scrapings (7)	15 843 000	25 800 000	Van Metre et al. 2012
	CT‐sealed pavement dust (11)	4 817 000	11 300 000	Mahler et al. 2010
	Gasoline exhaust/soot (2)	993 000	1 465 000	Boonyatumanond et al. 2007
	Diesel exhaust/soot (7)	116 000	671 000	Boonyatumanond et al. 2007
	Tire particles (6)	106 000	226 000	Boonyatumanond et al. 2007; Rogge et al. 1993
	Road dust (1)	58 700	58 700	Rogge et al. 1993
	Traffic tunnel dust (5)	22 600	25 000	Oda et al. 2001
	Unsealed asphalt pavement dust (7)	17 200	48 700	Mahler et al. 2010
	Brake lining particles (1)	16 200	16 200	Rogge et al. 1993
	Wood combustion (4)	14 100	29 700	Rogge et al. 1998; Schauer et al. 2001
	Concrete parking lot dust (2)	11 400	15 100	Mahler et al. 2010
	Asphalt (12)	11 100	28 000	Ahrens and Depree 2010; Boonyatumanond et al. 2007
	Asphalt‐sealed pavement dust (3)	8500	10 900	Mahler et al. 2010
Liquids	CT sealant product (1)	30 900 000	30 900 000	Van Metre et al. 2012
	Motor oil, used (9)	610 000	1 295 000	Boonyatumanond et al. 2007; Wong and Wang 2001
	Motor oil, unused (1)	2600	2600	Wong and Wang 2001

CT = coal tar.

### Mass fractions

Mass fractions analysis has been used as a tool to eliminate potential PAH sources as the primary source to environmental samples because of unlikely or even impossible mass fractions of source material required to achieve environmental PAH concentrations (Ahrens and Depree 2010; Baldwin et al. 2017).

The mass fraction for each sample/source combination was calculated by dividing the ΣPAH_16_ concentrations in samples by mean PAH concentrations of potential sources gathered from the literature (Table 3). The ΣPAH_16_ concentrations were used (rather than ΣPAH_36_) because ΣPAH_16_ is commonly reported in the literature, and thus the number of source concentrations included in the analysis could be maximized. The mass fraction therefore represented the hypothetical percentage mass of source material in a given sediment sample, assuming negligible contributions from other sources. We also assumed that PAHs are confined to the organic carbon fraction of the sediment sample, thereby providing an upper limit on the amount of source material (in percentage mass) possible in a given sample. If the source mass fraction was greater than the percentage of TOC in the sample, it was determined that the source could not possibly be the primary contributor of PAHs to the sediment (Table 4). If the mass fraction was <TOC but >½TOC, it was determined that the source was possible but unlikely to be the primary contributor of PAHs to the sediment, based on the assumption that >50% of the mass of organic carbon in a sediment sample is likely comprised of materials other than PAHs. If the mass fraction was <½TOC, it was determined that the source could be a primary contributor of PAHs to the sediment.

**Table 4:** Example mass fraction (MF) analysis for 3 scenarios with varying source concentrations

ΣPAH_16_ (µg/kg)		MF	TOC	MF, TOC relation	Can source be primary PAH contributor?
Sediment	Source				
1000	10 000	10%	2.0%	TOC < MF	Impossible
1000	70 000	1.4%	2.0%	TOC > MF > ½ TOC	Unlikely
1000	1 000 000	0.1%	2.0%	½ TOC > MF	Possible

ΣPAH_16_ = sum concentration of US Environmental Protection Agency 16 priority pollutant polycyclic aromatic hydrocarbon compounds; TOC = total organic carbon.

## RESULTS

### Observed concentrations relative to sediment quality guidelines

**Figure 2:**
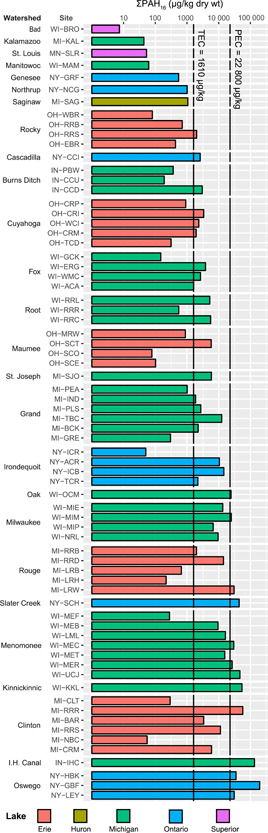
Sum concentrations of US Environmental Protection Agency 16 priority pollutant polycyclic aromatic hydrocarbon compounds (ΣPAH_16_) in streambed sediment samples. Site abbreviations are defined in Table 1. Watersheds are ordered by maximum PAH concentration; sites are ordered upstream to downstream within each watershed. PEC = consensus‐based probable effect concentration; TEC = consensus‐based threshold effect concentration.

Concentrations of ΣPAH_16_ in streambed sediment ranged from 7.4 to 196 000 µg/kg (mean 13 300 µg/kg; median 2600 µg/kg; Figure 2 and Table 5). Concentrations were especially high at 2 sites, NY‐GBF and IN‐IHC, where ΣPAH_16_ was >2 × higher than at other sites. The percentage of TOC ranged from 0.1 to 8.8, with a mean and median of 1.7 and 1.2, respectively. All PAH and TOC results are provided in the Supplemental Data, Table S6.

**Table 5:** Polycyclic aromatic hydrocarbons (PAHs) and toxicity quotients for individual samples

Site abbreviation	TOC (%)	ΣPAH_16_ (µg/kg)	Parent/alkyl ratio	HMW/LMW ratio	PECQ	TECQ	ΣESBTU
IN‐CCD	1.2	3030	2.1	3.6	0.1	1.9	0.4
IN‐CCU	0.8	191	0.2	1.1	0.0	0.1	0.1
IN‐IHC	6.8	135 000	0.6	1.4	5.9	83.6	5.0
IN‐PBW	0.5	367	1.7	5.7	0.0	0.2	0.1
MI‐BAR	0.5	3360	2.0	6.1	0.2	2.1	1.0
MI‐BCK	0.1	2240	4.8	7.1	0.1	1.4	3.7
MI‐CLT	0.1	294	3.0	7.3	0.0	0.2	0.3
MI‐CRM	1.4	5950	3.6	8.5	0.3	3.7	0.6
MI‐GRE	NA	303	1.7	7.2	0.0	0.2	NA
MI‐IND	0.2	1860	3.1	10.1	0.1	1.2	1.4
MI‐KAL	0.2	44.0	1.8	6.9	0.0	0.0	0.0
MI‐LRB	1.4	664	2.0	5.6	0.0	0.4	0.1
MI‐LRH	0.6	219	0.3	0.5	0.0	0.1	0.2
MI‐LRW	0.9	30 600	4.1	4.6	1.3	19.0	5.1
MI‐NBC	0.3	55.0	0.4	1.4	0.0	0.0	0.1
MI‐PEA	1.6	1010	2.5	5.3	0.0	0.6	0.1
MI‐PLS	0.1	2730	4.2	5.8	0.1	1.7	3.8
MI‐RRB	1.2	1990	2.1	4.6	0.1	1.2	0.3
MI‐RRD	0.7	14 200	3.8	5.1	0.6	8.8	2.9
MI‐RRR	2.3	57 600	3.8	12.1	2.5	35.8	3.5
MI‐RRS	0.4	11 400	3.7	6.1	0.5	7.1	3.9
MI‐SAG	2.2	1060	1.8	6.1	0.1	0.7	0.1
MI‐SJO	0.8	5890	2.1	6.0	0.3	3.7	1.1
MI‐TBC	0.4	12 400	4.7	4.5	0.6	7.7	5.2
MN‐SLR	1.4	53.0	0.5	1.6	0.0	0.0	0.0
NY‐ACR	0.4	10 500	3.4	4.6	0.5	6.5	3.7
NY‐CCI	1.4	2600	1.0	3.6	0.1	1.6	0.3
NY‐GBF	2.8	196 000	3.8	4.5	8.6	121.6	10.5
NY‐GRF	1.1	544	0.7	3.5	0.0	0.3	0.1
NY‐HBK	3.9	35 200	2.6	5.1	1.5	21.9	1.4
NY‐ICB	5.1	14 500	3.8	5.4	0.6	9.0	0.4
NY‐ICR	0.3	50.0	1.1	4.3	0.0	0.0	0.0
NY‐LEY	5.0	30 800	2.4	5.4	1.4	19.1	1.0
NY‐NCG	0.7	1010	2.5	10.0	0.0	0.6	0.2
NY‐SCH	2.0	44 100	4.1	8.0	1.9	27.4	3.1
NY‐TCR	0.3	2200	2.4	5.7	0.1	1.4	1.1
OH‐CRI	0.6	3360	3.5	1.1	0.2	2.1	1.0
OH‐CRM	0.4	1940	2.4	7.4	0.1	1.2	0.7
OH‐CRP	0.1	916	2.9	3.9	0.0	0.6	1.0
OH‐EBR	0.7	433	0.2	0.3	0.0	0.3	0.4
OH‐MRW	0.4	887	1.1	4.0	0.0	0.6	0.4
OH‐RRB	1.9	708	0.2	0.1	0.0	0.4	0.4
OH‐RRS	1.4	2010	0.6	0.9	0.1	1.3	0.5
OH‐SCE	1.0	102	0.3	0.6	0.0	0.1	0.1
OH‐SCO	0.5	78.0	0.4	1.8	0.0	0.1	0.1
OH‐SCT	1.4	5790	2.1	4.1	0.3	3.6	0.7
OH‐TCD	0.6	315	0.3	0.5	0.0	0.2	0.2
OH‐WBR	0.5	81.0	0.2	0.3	0.0	0.1	0.1
OH‐WCI	1.9	2360	0.9	1.1	0.1	1.5	0.3
WI‐ACA	1.8	1620	2.9	13.9	0.1	1.0	0.1
WI‐BRO	0.3	7.4	2.4	13.9	0.0	0.0	0.0
WI‐ERG	2.5	3840	2.3	7.6	0.2	2.4	0.2
WI‐GCK	0.3	149	2.6	11.2	0.0	0.1	0.1
WI‐KKL	2.4	54 200	3.5	5.1	2.4	33.7	3.5
WI‐LML	3.8	16 300	1.8	4.5	0.7	10.1	0.7
WI‐MAM	1.1	62.0	0.5	1.4	0.0	0.0	0.0
WI‐MEB	2.8	9530	2.9	5.9	0.4	5.9	0.5
WI‐MEC	2.0	29 900	3.5	5.9	1.3	18.6	2.2
WI‐MEF	8.8	281	1.2	4.2	0.0	0.2	0.0
WI‐MER	2.1	26 500	3.4	6.4	1.2	16.5	1.8
WI‐MET	2.3	15 500	3.4	5.6	0.7	9.6	1.0
WI‐MIE	2.1	13 400	2.0	4.6	0.6	8.3	1.0
WI‐MIM	5.1	25 000	2.4	5.6	1.1	15.5	0.8
WI‐MIP	7.0	6680	2.7	8.0	0.3	4.2	0.1
WI‐NRL	0.7	9550	3.6	11.9	0.4	5.9	1.8
WI‐OCM	1.6	24 500	2.8	4.9	1.1	15.2	2.3
WI‐RRC	4.2	5560	2.3	7.0	0.2	3.5	0.2
WI‐RRL	0.7	5230	2.9	7.3	0.2	3.3	1.2
WI‐RRR	1.1	548	1.9	8.0	0.0	0.3	0.1
WI‐UCJ	2.1	46 800	4.0	5.6	2.1	29.1	3.3
WI‐WMC	2.0	2670	2.0	5.7	0.1	1.7	0.2

Site abbreviations are defined in Table 1. TOC = total organic carbon; ΣPAH_16_ = sum concentration of US Environmental Protection Agency 16 priority pollutant PAH compounds; HMW = high molecular weight; LMW = low molecular weight; PECQ = consensus‐based probable effect concentration quotient; TECQ = consensus‐based threshold effect concentration quotient; ΣESBTU = sum equilibrium partitioning sediment benchmark toxicity units; NA = not measured or computed.

The TEC was exceeded in 62% of samples, with a median TECQ of 1.6 (Table 5). The PEC was exceeded in 18% of samples, with a median PECQ of 0.1. The highest PECQs were at NY‐GBF (8.6) and IN‐IHC (5.9). The ΣESBTU benchmark value of 1.0 was exceeded in 38% of samples, with a median ΣESBTU of 0.4. The highest ΣESBTUs were at NY‐GBF (10.5), Tributary to Buck Creek at Wyoming, MI (MI‐TBC; 5.2), and Lower River Rouge at Wayne, MI (MI‐LRW; 5.1).

### Identification of PAH sources

#### Land‐use analysis

The 6 urban land‐use categories (percentage area of parking lot, major transportation, commercial, industrial/military, residential, and urban total), percentage impervious, and population density were all significantly related to sediment ΣPAH_16_ concentrations (*p* < 0.05). Population density was the most strongly correlated with ΣPAH_16_ concentrations, with a Spearman's rank correlation coefficient (*r*) of 0.66 (Supplemental Data, Figure S1), followed by parking lot land use (*r* = 0.64), percentage impervious (*r* = 0.63), commercial land use (*r* = 0.62), and total urban land use (*r* = 0.61). The categories least correlated with ΣPAH_16_ concentrations were residential land use (*r* = 0.55), industrial/military land use (*r* = 0.48), and major transportation land use (*r* = 0.48).

#### Parent/alkylated and HMW/LWM compounds

The ratio of parent to alkylated compounds was >1.0 in 78% of the 71 streambed sediment samples, indicating a pyrogenic PAH source (Neff et al. 2005). The median ratio of parent/alkylated compounds was 2.4 across all sites (Table 5).

The HMW compounds were dominant over the LMW compounds in 90% of the streambed sediment samples, another indicator of a dominantly pyrogenic source of PAHs to most samples (Crane 2014). The median ratio of HMW/LMW compounds was 5.4 across all sites.

Seven samples had HMW/LMW and parent/alkylated ratios <1.0. Unlike the majority of the samples in the present study, the primary source of PAHs to these 7 samples was likely petrogenic. Four of the 7 samples were from the Rocky River basin (OH‐WBR, OH‐RRB, OH‐RRS, and OH‐EBR) where especially thin zones of depositional sediment may have resulted in inadvertent sampling of bank material; the remaining 3 samples were from the Cuyahoga (OH‐TCD), Maumee (OH‐SCE), and Rouge (MI‐LRH) River basins. The ΣPAH_16_ concentrations of these samples were generally low, ranging from 81.0 to 2010 µg/kg (median 315 µg/kg).

#### PAH profiles

**Figure 3:**
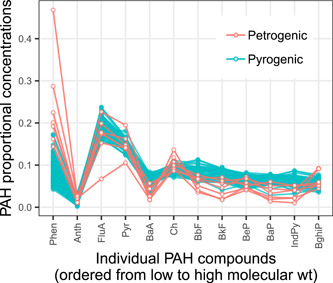
Comparison of polycyclic aromatic hydrocarbon (PAH) profiles in streambed sediments at sites with concentrations above detection levels (*n* = 70). Samples were classified as pyrogenic if the ratio of parent/alkyl compounds was ≥1.0 and/or the ratio of high‐molecular weight/low‐molecular‐weight (HMW/LMW) compounds was ≥1.0. Phen = phenanthrene; Anth = anthracene; FluA = fluoranthene; Pyr = pyrene; BaA = benz[*a*]anthracene; Ch = chrysene; BbF = benzo[*b*]fluoranthene; BkF = benzo[*k*]fluoranthene; BeP = benzo[*e*]pyrene; BaP = benzo[*a*]pyrene; IndPy = indeno[1,2,3‐*cd*]pyrene; BghiP = benzo[*g*,*h*,*i*]perylene.

The 12‐compound PAH profiles of the individual streambed sediment samples were generally similar, especially among those samples with a pyrogenic signature (i.e., those with ratios of parent/alkyl compounds ≥1.0 and/or HMW/LMW compounds ≥1.0; Figure 3 and Table 5). Profiles were variable among the 7 petrogenic samples, but were generally characterized by higher proportional concentrations of phenanthrene and lower proportional concentrations of benzo[*b*]fluoranthene, benzo[*k*]fluoranthene, benzo[*a*]pyrene, and indeno[1,2,3‐*cd*]pyrene.

**Figure 4:**
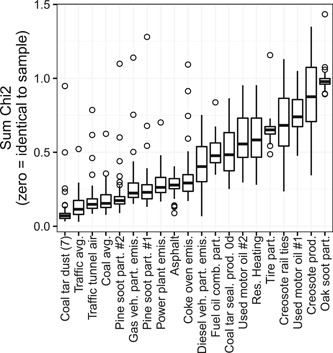
Chi‐square statistics between the 12‐compound profiles of streambed sediment samples and those of potential polycyclic aromatic hydrocarbon sources from the literature. Smaller χ^2^ statistics correspond to greater similarity. Boxes = 25th to 75th percentiles; dark line = median; whiskers = 1.5 × the interquartile range (IQR); circles = value outside the 1.5 × the IQR.

Comparisons with 12‐compound PAH source profiles from the literature showed that sediment samples were most similar to the profile of coal‐tar–sealed pavement dust (CTD7), with a median χ^2^ statistic of 0.07 (Figure 4). Other sources with profiles similar to sediment samples included vehicle/traffic average (VAVG; median χ^2^ 0.11), traffic tunnel air (TUN1; median χ^2^ 0.15), coal combustion average (CCB1; median χ^2^ 0.15), and pine combustion #2 (PIN2; median χ^2^ 0.17). Sources with profiles least similar to sediment samples included oak combustion (OAKS; median χ^2^ 0.98), creosote product (CRE4; median χ^2^ 0.88), used motor oil #1 and 2 (UMO1 and 2, median χ^2^ 0.74 and 0.56, respectively), creosote‐treated railway ties (CRE2; median χ^2^ 0.68), tire particles (TIRE; median χ^2^ 0.65), and residential heating (RESI; median χ^2^ 0.58).

#### PCA

**Figure 5:**
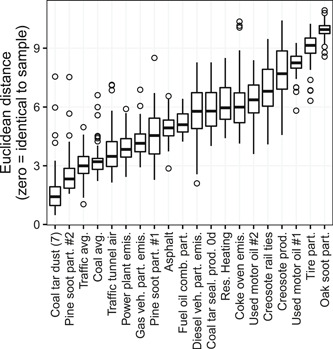
Euclidean distances between sources and samples for principal component analysis components 1 through 4 using 12‐compound polycyclic aromatic hydrocarbon (PAH) profiles. Boxes = 25th to 75th percentiles; dark line = median; whiskers = 1.5 × the interquartile range (IQR); circles = value outside the 1.5 × the IQR.

Principal components 1 to 4 each explained >10% of the total variance in the dataset and together explained 80% of the total variance. Consistently, Euclidean distances between streambed sediment samples and PAH sources (computed for all paired combinations of principal components 1–4; plotted in the Supplemental Data, Figure S2), identified coal‐tar–sealant pavement dust (CTD7) as the most similar source to streambed sediment samples (Figure 5). Other sources showing similarity to streambed sediment samples in some principal component combination graphs were vehicle/traffic average (VAVG), pine combustion #2 (PIN2), and coal combustion average (CCB1). Sources most distant from streambed sediment samples (i.e., largest Euclidean distances) were creosote‐treated railway ties (CRE2), creosote product (CRE4), used motor oil #1 (UMO1), tire particles (TIRE), and oak combustion (OAKS).

#### PMF receptor model

All PMF model runs converged in each of the 2‐, 3‐, and 4‐factor solutions using both default and relaxed convergence criteria (Supplemental Data, Table S7). The 3‐factor solutions (with default and relaxed convergence criteria) maximized the number of factors without violating model diagnostics recommendations described in the USEPA PMF User Guide (Norris et al. 2014) and listed in the Supplemental Data, Table S7. Relaxing the convergence criteria had little impact on the diagnostics of the 3‐factor solution (Supplemental Data, Table S7), so the default convergence criteria were used in the final solution. Scaled residuals plotted against ΣPAH_29_ indicated no bias in the goodness‐of‐fit of the final solution (Supplemental Data, Figure S5).

The proportional concentration profiles of factors 1 to 3 in the final 3‐factor PMF solution were generally similar to one another (Supplemental Data, Figure S6), with some exceptions noted below. Factor 1 contributed 48% of the total PAHs. The 12‐compound profile of factor 1 was similar to 2 sources, coal‐tar–sealed pavement dust (CTD7; χ^2^ 0.064) and vehicle/traffic average (VAVG; χ^2^ 0.069; Table 6). Because the χ^2^ value of coal‐tar–sealed pavement dust was only slightly lower than that of vehicle/traffic average (VAVG; χ^2^ difference of 0.005), factor 1 is interpreted to be a mixture of the 2 sources (and possibly others). Factor 2 contributed 31% of the total PAHs and, compared with factor 1, had slightly higher proportions of HMW compounds (Supplemental Data, Figure S6). The 12‐compound profile of factor 2 was most similar to that of coal‐tar–sealed pavement dust (CTD7; χ^2^ 0.133), followed by traffic tunnel air (TUN1; χ^2^ 0.163; Table 6). Compared with factor 1, the wider gap (0.03) in χ^2^ values between the top 2 sources lends more confidence in attributing factor 2 to a single source: coal‐tar–sealed pavement dust. Factor 3 contributed 21% of the total PAHs and, compared with factors 1 and 2, had higher proportional concentrations of phenanthrene and alkylated phenanthrenes/anthracenes. The 12‐compound profile of factor 3 was most similar to that of coal‐tar–sealed pavement dust (CTD7; χ^2^ 0.042) followed by vehicle/traffic average (VAVG; χ^2^ 0.082; Table 6). As with factor 2, the relatively large difference (0.04) in χ^2^ values between the top 2 sources provides some confidence in attributing factor 3 to coal‐tar–sealed pavement dust. Because the χ^2^ analysis of factors 2 and 3 attributed both factors to coal‐tar–sealed pavement dust, their contributions were combined. Thus, based on the PMF results, coal‐tar–sealed pavement dust was the dominant PAH source to 70% of samples (excluding samples omitted from the PMF analysis), contributing an average of 68% of the total PAHs to each sample (minimum 16%, median 68%, maximum 100%). The rest of the PAHs were from a mixture of sources, including coal‐tar–sealed pavement dust and vehicle/traffic average.

**Table 6:** Chi‐square (*χ* ^2^) statistics between 3‐factor unconstrained positive matrix factorization solutions and 12‐compound source profiles from the literature^a^

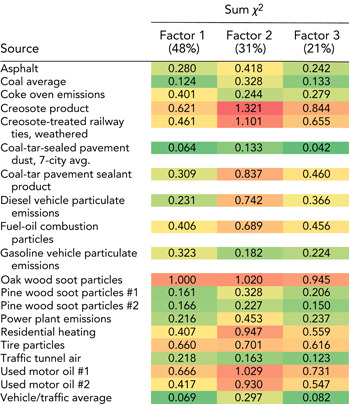

^a^ Factor percentages are the percentage of total polycyclic aromatic hydrocarbons. The color gradation (from most similar to least similar) is dark green‐light green‐yellow‐orange‐red.

**Figure 6:**
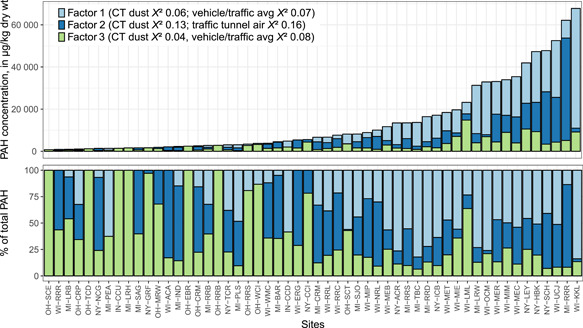
The estimated contribution of polycyclic aromatic hydrocarbons (PAHs) from different sources to individual sediment samples. Contributions are based on the 3‐factor positive matrix factorization (PMF) model. Source identities are based on similarity—determined using the chi‐square statistic—between PMF factor profiles and 12‐compound source profiles from the literature (Table 2 and Supplemental Data, Table S3). For site abbreviations, see Table 1. CT dust = coal‐tar‐sealed pavement dust.

The χ^2^ statistics between PAH sources and some of the other model runs (i.e., the 3‐factor model with relaxed convergence criteria, and the 2‐factor models with default and relaxed convergence criteria) are provided in the Supplemental Data, Table S8. Contributions to individual sediment samples were estimated for each PMF factor in the final 3‐factor solution (Figure 6).

#### Mass fractions

**Figure 7:**
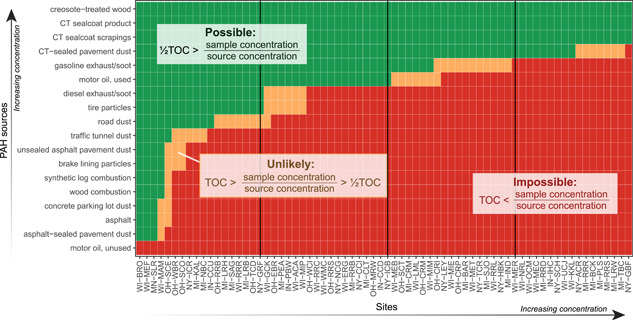
The likelihood of different polycyclic aromatic hydrocarbon (PAH) sources to be the primary source to individual streambed sediment samples, based on PAH concentration and total organic carbon (TOC) in samples versus PAH concentration in sources. Source concentrations are means of up to 12 samples compiled from previous studies (Supplemental Data, Table 3). Sites are ordered by TOC‐normalized PAH concentration. CT = coal tar. For site abbreviations, see Table 1.

For most sediment samples, mass fractions analysis considerably narrowed the list of potential primary PAH sources. Many potential primary PAH sources to streambed sediment samples had PAH concentrations in the lowest approximately 10th percentile (Figure 7), but the number of potential primary sources diminished rapidly with increasing sample concentrations. The PAH sources such as asphalt‐sealed pavement dust, wood combustion, and traffic tunnel and road dust could not be the primary sources to the upper approximately 75% of sediment samples (by total PAH concentration) because the source PAH concentrations were not high enough. For the top approximately 25% of sediment samples, only the most concentrated PAH sources remained as potential primary sources: creosote‐treated wood and coal tar (as sealcoat product, pavement scrapings, or pavement dust; or as historical coal tar contamination from former manufactured gas plants).

## DISCUSSION

**Figure 8:**
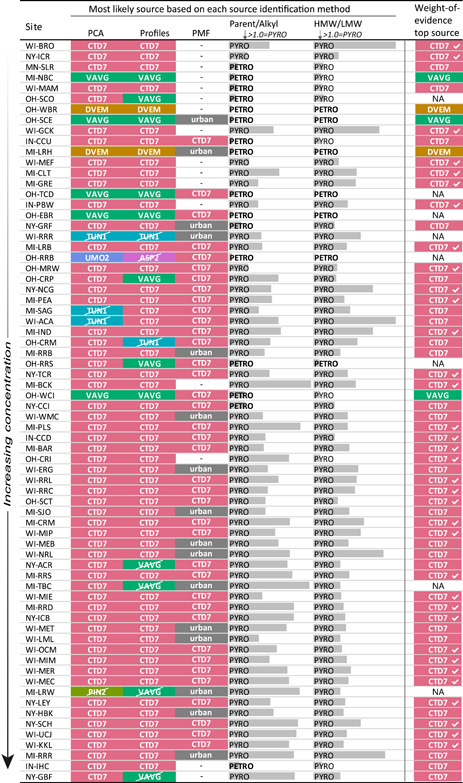
Synthesis of conclusions from different polycyclic aromatic hydrocarbon source identification methods. The sources determined to be the likely primary sources to each site using principal component analysis (PCA), profiles analysis, and the positive matrix factorization (PMF) model are listed. For PMF, the dominant source was coal‐tar–sealed pavement dust (CTD7) where the sum contribution of factors 2 and 3 exceeded 50%, and the dominant source was “mix” where the contribution of factor 1 exceeded 50%. Sources determined to be impossible based on mass fractions analysis are struck‐through. Gray bars indicate parent/alkyl ratios and high‐molecular‐weight to low‐molecular‐weight (HMW/LMW) ratios, with values <1.0 indicative of a petrogenic (PETRO) source, and values >1.0 indicative of a pyrogenic (PYRO) source. The “weight‐of‐evidence top source” (right column) is identified where >50% of the identification methods agree, with a check indicating unanimous agreement. NA = ≤50% of identification methods agree. Site MI‐KAL is omitted because of concentrations below the detection limit. Site abbreviations are defined in Table 1. VAVG = vehicle/traffic average; DVEM = diesel vehicle particulate emissions; TUN1 = traffic tunnel air; PIN2 = pine wood soot particles; UMO2 = used motor oil; ASP2 = asphalt.

Multiple lines of evidence were used to determine the most likely source of PAHs to sediment samples from Great Lakes tributaries. The results of the individual source identification methods for each site are summarized in Figure 8. The likely dominant sources to each site determined by using PCA, profiles analysis, and the PMF model are listed. For PMF, the dominant source was coal‐tar–sealed pavement dust (CTD7) where the sum contribution of factors 2 and 3 exceeded 50%, and the dominant source was a “mix” where the contribution of factor 1 exceeded 50%. Sources determined to be impossible based on mass fractions analysis are struck‐through. Gray bars indicate parent/alkyl ratios and HMW/LMW ratios, with values <1.0 indicative of a petrogenic source, and values >1.0 indicative of a pyrogenic source. For samples with majority agreement (i.e., >50% of the identification methods agree) between the multiple lines of evidence, the most likely primary PAH source is identified in the rightmost column (“weight‐of‐evidence top source”). The weight‐of‐evidence top source has a check mark for samples with unanimous agreement across the multiple lines of evidence, indicating greater confidence. Some of these lines of evidence are more powerful than others; although assigning weights to them would be subjective, the weakest lines of evidence are likely the parent/alkyl and HMW/LMW ratios, and the most powerful is likely mass fractions.

For 35 of the sampled sites (49%), unanimous agreement across all lines of evidence indicated that coal‐tar–sealed pavement dust was the most likely primary source of PAHs. At an additional 22 sites (31%), coal‐tar–sealed pavement dust was identified as the most likely primary source by the majority of (but not all) methods. At some sites, some portion of the coal‐tar signature may have been from former manufactured gas plant contamination. Sampling surficial sediment from tributary streambeds was meant to minimize historical contributions, but it is possible that historical sediments were reworked and deposited at the surface.

Vehicle emission‐related sources (VAVG and DVEM) were identified as the most likely sources at 5 sites (10%). At 8 sites there was no majority agreement on the most likely primary source. Creosote, despite having a very high PAH concentration, was not identified as the likely primary source at any site because its unique PAH profile differed considerably from the profiles of sediment samples. Likewise, coal combustion, a common PAH source in urban areas, was not identified as a primary source at any site.

The land‐use analysis lends some support to the conclusion that coal‐tar–sealed pavement dust was the most likely primary source of PAHs. Parking land use was found to better correlate with sediment PAH concentrations than major transportation land use (i.e., major roads), despite the similarities between these 2 categories: they are impervious surfaces made from materials that accumulate tire and brake particles, motor oil, exhaust from diesel and gasoline engines, and atmospheric deposition of PAHs. An important difference between parking areas and major roads is that pavement sealants are commonly used on parking areas (if made of asphalt) but are not typically used on major roads.

The methods used in the present study provide an evidence‐based approach for identifying the most likely sources contributing to each sample, but each method has limitations and uncertainties including PAH source and sediment concentrations, inability of source profiles from the literature to capture variability in PAH sources in the study region (some of the source profiles are decades old or from other countries), variability in data quality in literature source profiles, the potential misinterpretation of results if the analysis lacks an important PAH source, and the potential for weathering to affect PAH profiles in sediment samples (Baldwin et al. 2017). The lack of consensus among the methods for 51% of the sites highlights these uncertainties and the value of using multiple lines of evidence, which mitigates uncertainties of individual methods and strengthens the overlapping conclusions (Larsen and Baker 2003; O'Reilly et al. 2014).

There is a need for an updated, comprehensive set of source profiles to improve future source‐identification studies. The present study used 12‐compound source profiles gathered from several different studies, with varying and often unknown measurement uncertainties. An updated list of source profiles, collected and analyzed using consistent methods, would provide a better understanding of uncertainties in the profiles and on the analyses reliant on the profiles. Including a greater number of compounds (≥16) may help differentiate between sources with similar profiles.

A limitation of our study was the assumption that a single sample was used to represent the PAH concentration and profile at each location. The PAH concentrations in streambed sediment are not spatially homogenous, as illustrated by the duplicate samples in the present study, which had median RPDs of up to 42.4% for individual compounds. However, despite the variability in concentrations between duplicate field samples, the PAH profiles of duplicate samples were quite similar (Supplemental Data, Figure S7). In fact, the PAH profiles were similar not only within a site, but at most of the sampled locations across the Great Lakes Basin (Figure 3). This finding suggests that, although multiple samples at each location would have shown a potentially wide range in concentrations, the PAH profiles may not have differed substantially.

**Figure 9:**
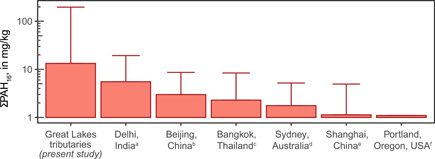
Comparison of the sum concentration of the 16 US Environmental Protection Agency priority pollutant polycyclic aromatic hydrocarbons (ΣPAH_16_) in urban sediments in select locations around the world. bar = mean; whisker = maximum. ^a^Kumar et al. 2016; ^b^Shen et al. 2009; ^c^Boonyatumanond et al. 2006; ^d^Nguyen et al. 2014; ^e^Yang et al. 2018; ^f^Yanagida et al. 2012.

Coal‐tar–sealed pavement dust has been identified as the likely primary source of PAHs to streambed sediments elsewhere in the central and eastern United States, including in urban and suburban lakes, streams, and stormwater ponds in Austin (TX; Mahler et al. 2005), Springfield (MO; Pavlowsky 2013), Fort Worth (TX; Yang et al. 2010), Durham (NH; Watts et al. 2010), Minnesota (Crane 2014), Milwaukee (WI; Baldwin et al. 2017), and other locations (Van Metre and Mahler 2010). The ubiquity of the coal‐tar–sealed pavement dust profile in sediments across the Great Lakes Basin and elsewhere raises the question of whether that profile may actually represent “urban background” (i.e., the mixture of common urban PAH sources such as coal and wood combustion and vehicle emissions). However, traditional urban background sources cannot account for the high PAH concentrations measured in many of the samples in our study. This point was demonstrated using mass fractions analysis and is further supported by simply comparing PAH concentrations from the present study with concentrations in areas where coal‐tar sealants are not used (i.e., outside of the eastern and central United States and Canada). Compared with a ΣPAH_16_ mean of 13 300 µg/kg and maximum of 196 000 µg/kg for the present study, studies of urban streams, canals, drains, and lakes in Portland (OR, USA; Yanagida et al. 2012), Sydney (Australia; Nguyen et al. 2014), Delhi (India; Kumar et al. 2016), Beijing (China; Shen et al. 2009), Shanghai (China; Yang et al. 2018), and Bangkok (Thailand; Boonyatumanond et al. 2006) reported mean ΣPAH_16+_ concentrations of 663 to 5570 µg/kg (Figure 9). The maximum ΣPAH_16+_ concentrations in these studies were all <9000 µg/kg, with the exception of Delhi storm drain sediments, which had a maximum of 19 300 µg/kg. Although not a comprehensive examination of PAH concentrations in urban sediments worldwide, these studies indicate that ΣPAH_16_ concentrations are typically <9000 µg/kg where the primary PAH sources are urban background related. Even in the end‐member case of Delhi storm drains, which likely approach the urban background maximum, ΣPAH_16_ concentrations do not exceed 20 000 µg/kg. Thirty‐two percent of the sites in the present study had ΣPAH_16_ concentrations >9000 µg/kg, and 18% had ΣPAH_16_ concentrations >20 000 µg/kg. Based on these comparisons, either 1) urban background PAH concentrations are substantially higher in the Great Lakes Basin than in these other locations in China, India, Thailand, and elsewhere; or 2) another PAH source, unrelated to traditional urban background sources and not present in these other locations, is elevating PAH concentrations above typical urban background concentrations in Great Lakes tributaries. A study of global atmospheric PAH emissions from coal, petroleum, and biofuel consumption and transformation (i.e., traditional urban background sources) reported an annual atmospheric PAH emission density (annual emission rate/land area) of 4.3 kg/km^2^/y^–1^ for the United States (excluding Alaska and Hawaii; Zhang and Tao 2009). In comparison, the reported atmospheric PAH emission density for China was 12.2 kg/km^2^/y^–1^, and for India 30.2 kg/km^2^/y^–1^. Given the much lower PAH emission density in the United States compared with China and India, it seems logical that sediments in the United States would tend to have lower rather than higher PAH concentrations. The fact that PAH concentrations in our study commonly exceeded the maximum concentrations measured in urban sediments in China and India suggests that a different PAH source, unrelated to traditional urban background sources and not widely present in these other locations, is elevating PAH concentrations in Great Lakes tributaries. Based on the multiple lines of evidence in the present and previous studies (Mahler et al. 2005; Van Metre and Mahler 2010; Watts et al. 2010; Yang et al. 2010; Pavlowsky 2013; Crane 2014; Baldwin et al. 2017; Valentyne et al. 2018), that source is most likely coal‐tar–sealed pavement dust.

## Supplemental Data

The Supplemental Data are available on the Wiley Online Library at https://doi.org/10.1002/etc.4727.

## Disclaimer

Any use of trade, product, or firm names is for descriptive purposes only and does not imply endorsement by the US Government. The views expressed in this article are those of the authors and do not necessarily represent the views or policies of the US Environmental Protection Agency. The US Environmental Protection Agency through the Office of Research and Development provided technical direction but did not collect, generate, evaluate, or use the environmental data described in the present study.

## Supporting information

This article includes online‐only Supplemental Data.

Supporting information

Supporting information

## Data Availability

Data are provided in the Supplemental Data and are also available online at https://doi.org/10.5066/F7P55KJN and at https://waterdata.usgs.gov/nwis.
