# Supplementary material for: Primary Sources of Polycyclic Aromatic Hydrocarbons to Streambed Sediment in Great Lakes Tributaries Using Multiple Lines of Evidence
Source: Environ Toxicol Chem. 2020 Jun 11;39(7):1392–408. doi: 10.1002/etc.4727 (PMC7383861; doi:10.1002/etc.4727)
Supplement: Supplementary file 2 — Supporting information [file ETC-39-1392-s002.docx]

*Supplemental Information to*

Primary sources of PAHs to Great Lakes tributaries using a multiple-lines-of-evidence approach

# Table of Contents

Sample collection and analysis methods....................................................................................................S1

**SI Figure S1.** Relations between streambed sediment total PAH_16_ concentrations and the percentage of each basin in different urban-related land uses.........................................................................................S5

**SI Figure S2.** Combinations of principle components 1-4 for 12-compound proportional concentration profiles of streambed sediment samples and potential PAH sources from the literature.........................S6

Positive matrix factorization model...........................................................................................................S7

**SI Figure S3.** Constrained versus unconstrained Positive Matrix Factorization species profiles for factors 1, 2, and 3, resulting from pulling factor 1 toward coal-tar-sealed pavement dust...................................S8

**SI Figure S4.** Constrained versus unconstrained Positive matrix factorization species profiles for factors 1, 2, and 3, resulting from pulling factor 1 toward vehicle/traffic average................................................S9

**SI Figure S5.** Scaled residuals of the final Positive matrix factorization model versus total PAH concentrations (ΣPAH_29_)............................................................................................................................S10

**SI Figure S6.** Positive matrix factorization species profiles for factors 1, 2, and 3...................................S10

**SI Figure S7.** Comparison of polycyclic aromatic hydrocarbons (PAH) profiles in duplicate and regular streambed sediment samples...................................................................................................................S11

## Sample collection and analysis methods

Streambed sediment sample collection was performed either by boat or while wading in the stream following USGS protocols (Shelton 2008). A WaterMark® Universal Core Head Sediment Sampler (Forestry Suppliers, Jackson, MS) outfitted with a 70 mm (2-3/4 inch) outer diameter by 66.7 mm (2-5/8 inch) inner diameter with 1.6 mm (1/16 inch) wall polycarbonate tubing (Forestry Suppliers, Jackson, MS; or United States Plastic Corp®, Lima, OH) was used for sediment collection^[[1]](#footnote-1)^. To prepare polycarbonate tubing for sample collection it was cut to 46 cm in length; at 16.5 cm from the bottom of the 46 cm core tube a 6 mm diameter hole was drilled. Polycarbonate tubes were then scrubbed with a brush using soapy water followed by three rinses each of tap water and deionized water prior to drying and bagging for transport to the sampling locations. An individual polycarbonate tube was prepared for each sampling location.

Prior to streambed sediment collection, electrical tape was used to cover the 6 mm drill hole in the polycarbonate tube. Once applied, the core tube, utensils, and a stainless-steel pan were thoroughly rinsed with native sampling location water prior to initial contact with the sediment surface. Once a depositional location with fine grain sediment was identified, streambed sediment was collected by pushing the Universal Core Head Sampler with polycarbonate tubing into the sediment more than 15 cm. The core was carefully removed from the sediment and brought above the water surface, inspected to ensure that a good sediment core with the flocculant sediment-water interface was still intact, and excess bottom sediment was removed to a point where there was 15 cm of sediment left in the polycarbonate tube. Electrical tape was removed from the 6 mm drill hole to allow the overlying water to drain without disruption to the sediment-water interface.

Sediment was removed from the polycarbonate tube directly into a stainless-steel pan while trying to maintain the sediment core structure. Using a stainless-steel spatula, the core was divided in half lengthwise from top to bottom. One of these divided sediment halves was placed into an amber glass 1 L bottle for use in the present study. The other half was used for a separate study. Samples were then capped and placed on ice for a maximum of 48 hours post collection until shipment to Battelle Memorial Institute laboratories in Norwell, MA. All sediment processing equipment (e.g. stainless-pan, spatulas, etc.) were field cleaned between each sampling location by scrubbing with soapy water followed by three rinses each of tap and deionized water.

Samples were analyzed for 18 parent and 18 alkylated PAHs by Battelle Memorial Institute in Norwell, MA (SI table S2). Methods followed Battelle SOP 5-157-17 *Identification and quantification of semi-volatile organic compounds by gas chromatography mass spectrometry* (GC/MS), which was based on EPA method 8270D modified (U.S. Environmental Protection Agency 1998); and Battelle SOP 5-191-07 *HPLC (GPC) cleanup of sample extracts for semivolatile organic pollutants*. The summary of methods below is taken from Battelle’s QA/QC Summary for this study. Additional details are available in the methods documents. Upon their arrival at the laboratory, samples were stored refrigerated until homogenized and split for analysis. An aliquot was used for chemical analysis and was frozen. The remainder was refrigerated and forwarded to ALS Environmental, Kelso, WA, for total organic carbon (TOC) analysis. Approximately 20 g of sediment was spiked with four surrogates and serially extracted three times with dichloromethane using an orbital shaker table. The combined extracts were concentrated by Kuderna-Danish and nitrogen evaporation techniques. Sample concentrates were further processed by alumina and copper cleanup, followed by size-exclusion gel-penneation chromatography (GPC) in combination with high pressure liquid chromatography (HPLC) (Waters Envirogel column setup, Waters 1515 pump, Waters 717 autosampler, Waters 2487 UV detector, Waters Fraction Collector III). Final extracts were fortified with internal standard (IS) compounds and submitted for PAH analysis by gas chromatography mass spectrometry (GC/MS; Agilent HP6890 Gas Chromatograph and HP5973A Mass Selective Detector, Agilent HP7683 autosampler, Agilent DB-5 capillary column). PAH analysis by GC/MS operated in selected ion monitoring (SIM) mode. An initial calibration of target analytes was analyzed prior to analysis to demonstrate the linear range of analysis. Calibration verification was performed at the beginning and end of each 24-hour period in which samples were analyzed. Concentrations of PAH were calculated versus internal standards using the average response factors (RF) generated from the initial calibration (EPA 8270D modified).

Four surrogate compounds were added to each environmental sample by Battelle prior to extraction: naphthalene-d8, acenaphthene-d10, phenanthrene-d10, and benzo(a)pyrene-d12. Recoveries of naphthalene-d8 ranged from 43 – 89% with a median of 59%. Similar recovery ranges and median values were noted for the remaining three compounds: 46 – 87%, median of 63% acenaphthene-d10; 40 – 88%, median of 60% benzo(a)pyrene-d12; and 46 – 94%, median of 69% phenanthrene-d10.

Battelle performed 11 procedural blanks, 5 matrix spikes, 5 lab duplicates, and 10 laboratory control spikes (LCS) over the course of five analytical batch analyses for this study. For the 11 procedural blank samples, there was a total of 396 PAH compound results from which 96% were below the detection limit. Of the 4% procedural blanks above the detection limit, only three compounds had exceedances: naphthalene (63%, 0.12 – 0.26 µg/kg), phenanthrene (31%, 0.08 – 0.15 µg/kg) and pyrene (6%, 0.12 µg/kg). All were flagged as “estimated” values. For the five matrix spikes, there were a total of 43 PAH compound results; percent recovery ranged from 44 – 117%, with a median of 73%. Batelle noted that “Several target analytes over all the analytical batch analyses had percent recoveries outside the measurement quality objective (MQO) range, however in each instance the concentration in the matrix spike was less than 5x the background concentration and therefore the results aren’t appropriate for data quality assessment.” Accordingly, there were no primary exceedances noted for the matrix spikes. The five lab duplicates’ relative percent difference (RDP) over all the compounds ranged from 0 to 197%, with a median of 26%. Over the course of the five analytical batches, there were 76 exceedances for duplicate precision, likely due to sample inhomogeneity. LCS percent recoveries ranged from 54 – 99%, median 79%.

TOC analysis was done using American Society for Testing and Materials (ASTM) D4129-05 modified method and a 0.05 percent reporting detection limit (RDL). All method blanks (n=5) were below the detection limit, and all duplicates (n=5) had relative percent differences < 4%. All samples for TOC analysis exceeded the recommended holding time, and the data were flagged appropriately.


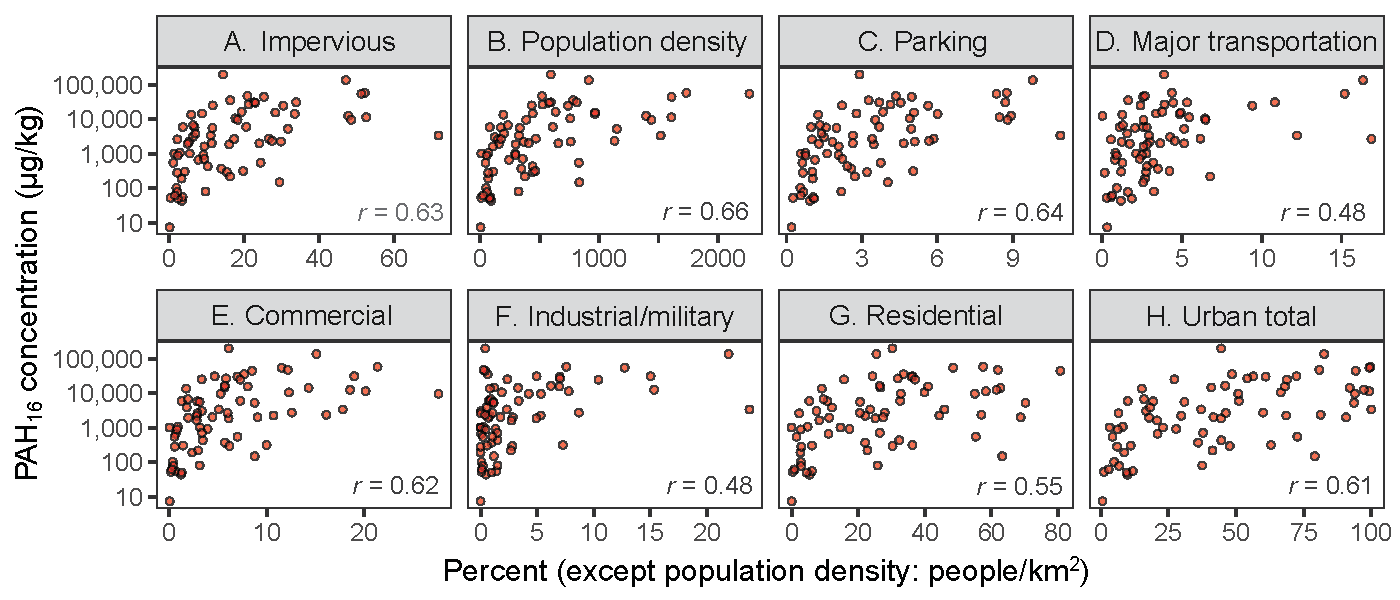


**SI Figure S1.** Relations between streambed sediment total PAH_16_ concentrations and the percentage of each basin in different urban-related land uses. *r* = Spearman correlation coefficient; PAH_16_ = 16 polycyclic aromatic hydrocarbon compounds.


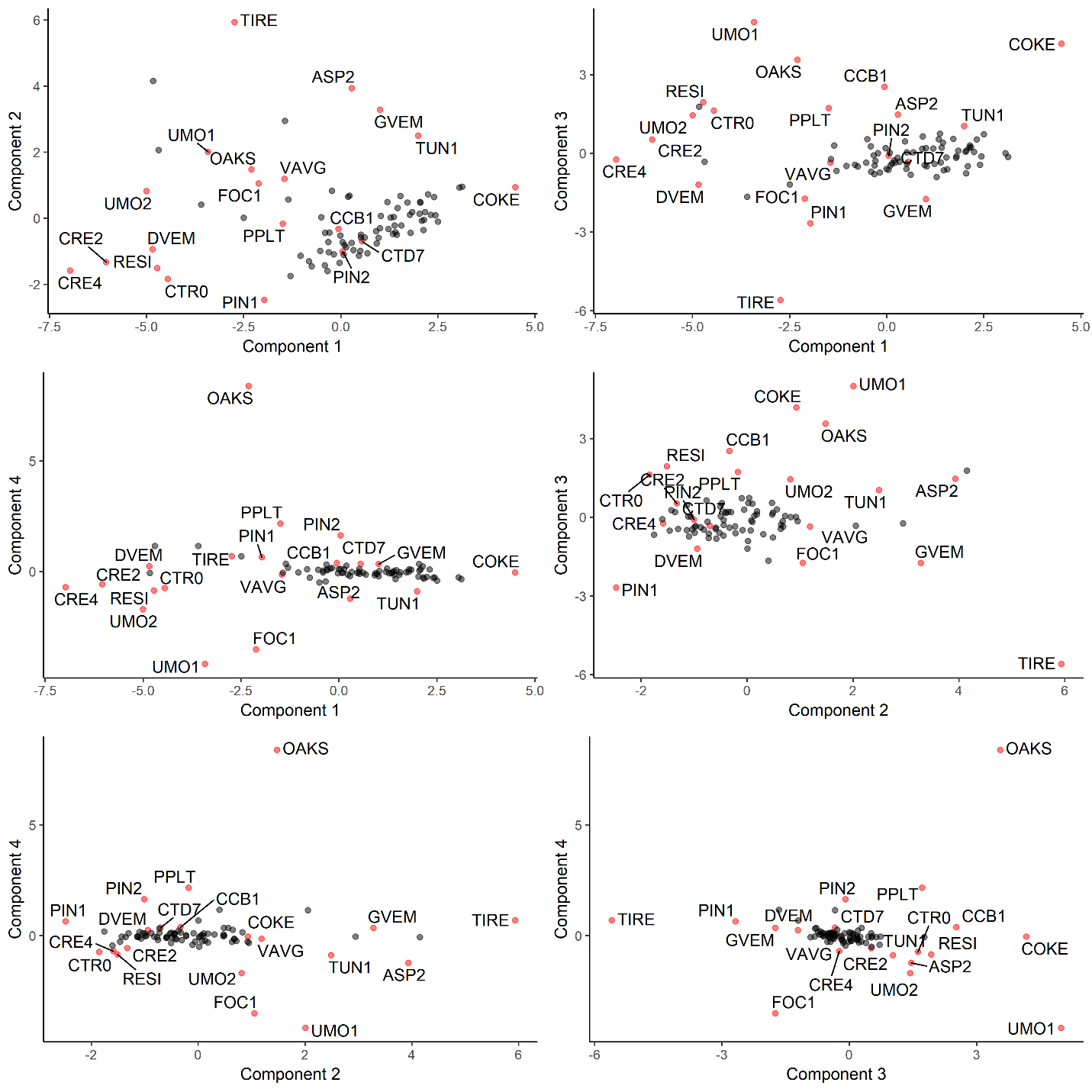


**SI Figure S2.** Combinations of principle components 1-4 for 12-compound proportional concentration profiles of streambed sediment samples (grey circles) and potential PAH sources from the literature (red circles). PAH source abbreviations are defined in Table 2.

# Positive matrix factorization model

PMF was run using two different sets of convergence criteria: one default and one “relaxed”. The convergence criteria were “relaxed” by adjusting the deltaQ values in the EPA-PMF file *PMF_bs_6f8xx_sealed_GUI.ini* using a text editor. Starting at line 1424, the default values of 0.1000, 0.0050, 0.0003 were relaxed to 5.000, 0.100, 0.010, respectively.

Constraints were applied to the final PMF solution to test rotational ambiguity and to try to “pull” factor 1 toward one or the other of the two sources most similar to factor 1: CTD7 and VAVG. To pull factor 1 toward CTD7, we used two ratios derived from the 12-compound source profile of CTD7: indeno[1,2,3-cd]pyrene / benzo[g,h,i]perylene = 0.96, and fluoranthene / pyrene = 1.35. The results of adding these constraints are shown in SI Figure S3. To pull factor 1 toward VAVG, which was done in a separate run, we used indeno[1,2,3-cd]pyrene / benzo[g,h,i]perylene = 0.77, and fluoranthene / pyrene = 0.98 (derived from the 12-compound source profile of VAVG). The results of adding these constraints are shown in SI Figure S4. Because the constrained and unconstrained solutions were similar, the unconstrained solution was used as the final model.


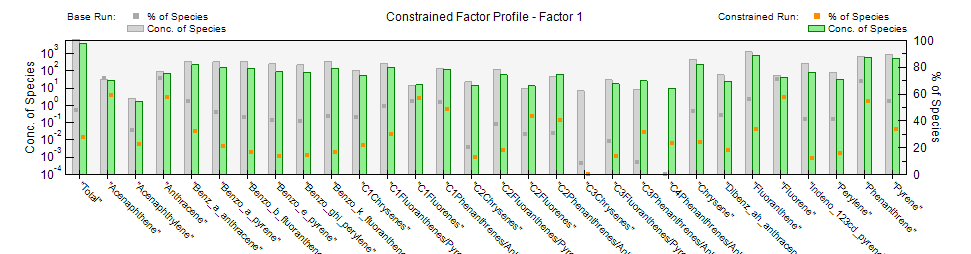


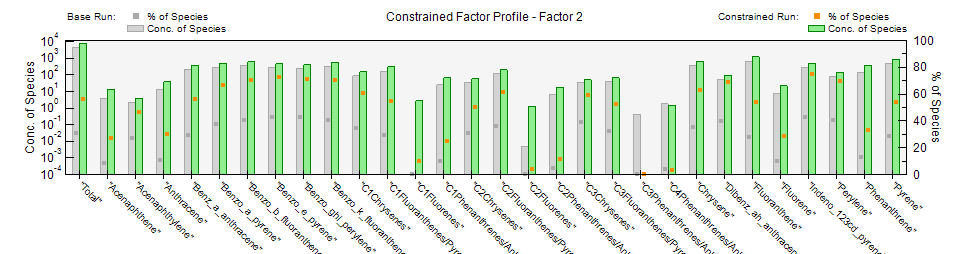


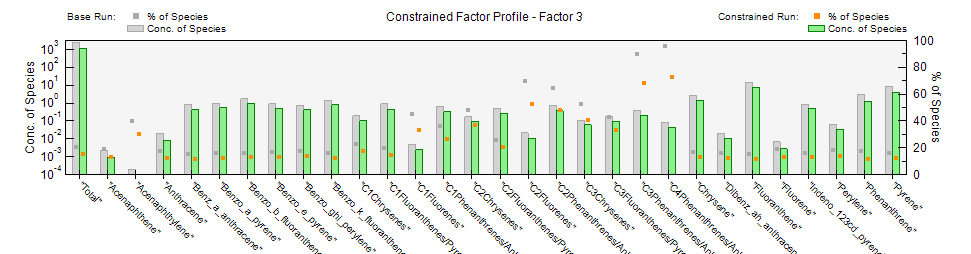


**SI Figure S3.** Constrained (orange, green) versus unconstrained (grey) positive matrix factorization species profiles for factors 1 (top), 2 (middle), and 3 (bottom), resulting from pulling factor 1 toward coal-tar-sealed pavement dust (CTD7).


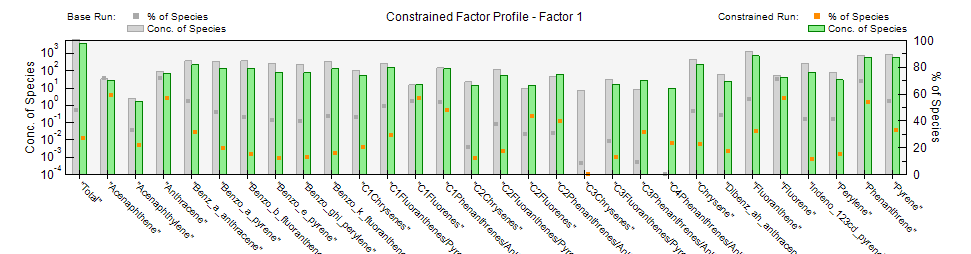


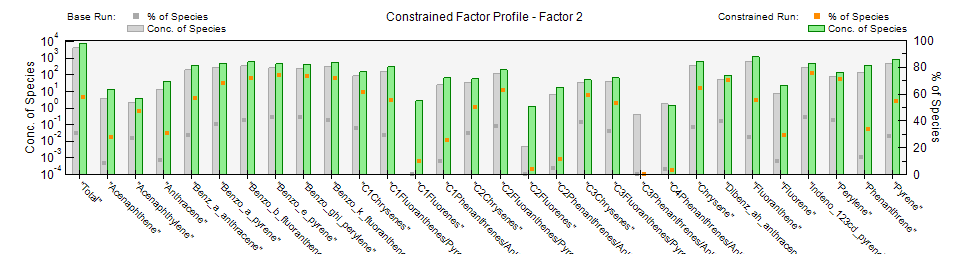


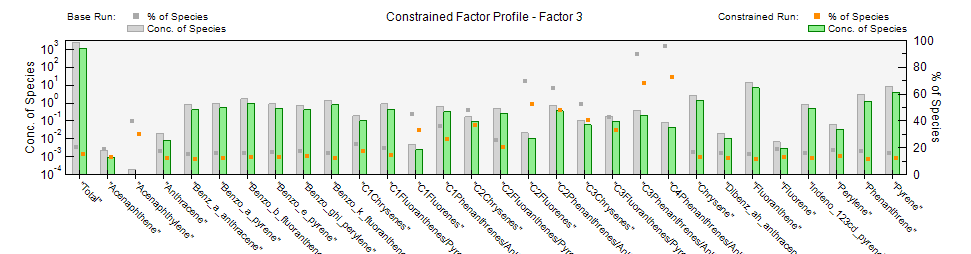


**SI Figure S4.** Constrained (orange, green) versus unconstrained (grey) positive matrix factorization species profiles for factors 1 (top), 2 (middle), and 3 (bottom), resulting from pulling factor 1 toward vehicle/traffic average (VAVG).

**SI Figure S5.** Scaled residuals of the final positive matrix factorization (PMF) model versus total PAH concentrations (ΣPAH_29_).


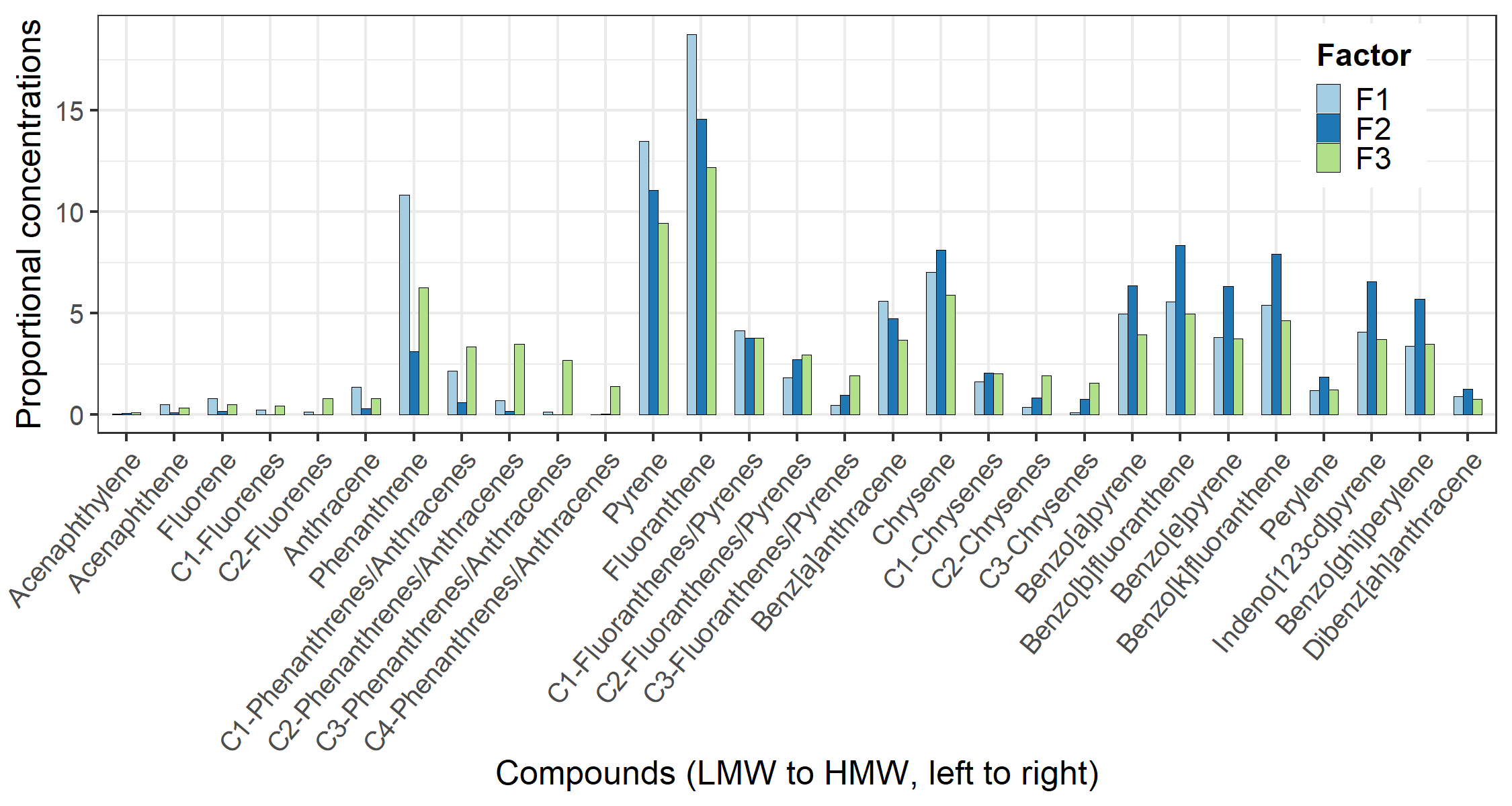


**SI Figure S6.** Positive matrix factorization species profiles for factors 1 (F1), 2 (F2), and 3 (F3).


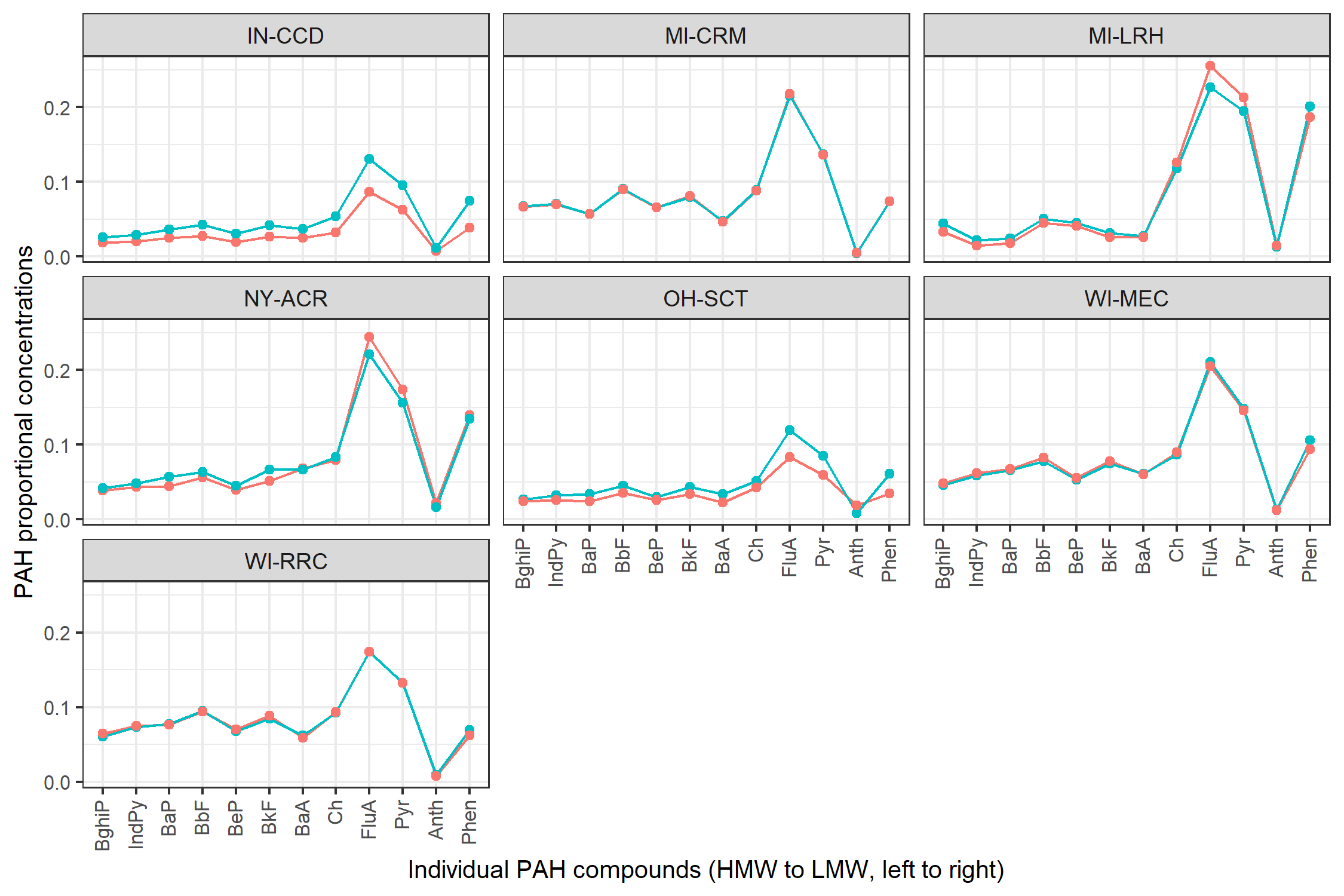


**SI Figure S7.** Comparison of polycyclic aromatic hydrocarbons (PAH) profiles in duplicate (red) and regular (blue) streambed sediment samples. Samples collected at WI-BRO omitted because of concentrations below the detection limit.

1. Any use of trade, firm, or product names is for descriptive purposes only and does not imply endorsement by the U.S. Government. [↑](#footnote-ref-1)
